# Ultra-broadband achromatic imaging with diffractive photon sieves

**DOI:** 10.1038/srep28319

**Published:** 2016-06-22

**Authors:** Xiaonan Zhao, Jingpei Hu, Yu Lin, Feng Xu, Xiaojun Zhu, Donglin Pu, Linsen Chen, Chinhua Wang

**Affiliations:** 1 College of Physics, Optoelectronics and Energy & Collaborative Innovation Center of Suzhou Nano Science and Technology, Soochow University, Suzhou 215006, China; 2 Key Lab of Advanced Optical Manufacturing Technologies of Jiangsu Province & Key Lab of Modern Optical Technologies of Education Ministry of China, Soochow University, Suzhou 215006, China; 3 School of Electronics and Information, Nantong University, Nantong 226019, Jiangsu, China

## Abstract

Diffractive optical elements suffer from large chromatic aberration due to the strong wavelength-dependent nature in diffraction phenomena, and therefore, diffractive elements can work only at a single designed wavelength, which significantly limits the applications of diffractive elements in imaging. Here, we report on a demonstration of a wavefront coded broadband achromatic imaging with diffractive photon sieves. The broadband diffraction imaging is implemented with a wavefront coded pinhole pattern that generates equal focusing power for a wide range of operating wavelength in a single thin-film element without complicated auxiliary optical system. Experimental validation was performed using an UV-lithography fabricated wavefront coded photon sieves. Results show that the working bandwidth of the wavefront coded photon sieves reaches 28 nm compared with 0.32 nm of the conventional one. Further demonstration of the achromatic imaging with a bandwidth of 300 nm is also performed with a wavefront coded photon sieves integrated with a refractive element.

Diffractive optical elements (DOEs) have increasingly shown a promising type of optical imaging components in modern optical systems, such as ultra-large space telescope primaries^1^, high-resolution microscopy^2^,^3^, spectroscopy^4^, THz optics for tomographic imaging^5^, X-ray or EUV lithography^6^ that are difficult, or even impossible, with conventional glass-based refractive optics, because of their unique characteristics of compact size, light weight, and high degree of design flexibility. Various Fresnel zone plates (FZP) are typical diffractive elements in which a series of concentric circular rings of equal area with alternating absorbing and transmitting zones. The focusing effect is created by the constructive interference of waves passing through the transmitting zones distributed periodically or non-periodically along the radial coordinate^7^,^8^,^9^. Photon sieves, which is evolved from the traditional FZP, is a new class of diffractive element in which the clear zones are replaced by great number of non-overlapping pinholes of different sizes^10^. The focusing properties of the photon sieves can be significantly improved in terms of sidelobe suppression and spatial resolution of focal point because of that apodization can be easily incorporated into a photon sieves simply by modifying the number of holes per zone^11^,^12^. Several theoretical and experimental studies on different types of the photon sieves were carried out, such as large aperture photon sieves^13^, multi-region photon sieves^14^. Very recently, Huang *et al*. proposed ultrahigh-capacity photon sieves operating at *λ* = 632.8 nm and experimentally demonstrated the accurate manipulation of light to realize a high diffraction efficiency hologram and a super-resolution focusing spot^15^.

However, all the imaging DOE suffers from large chromatic aberration due to the nature of very strong wavelength in diffraction phenomena, and therefore, all those mentioned DOEs can work only at a single designed wavelength, which significantly limits the applications of DOEs in imaging. Gimenez *et al*. showed that a fractal zone plate (FZP) exhibits an extended depth of field and a reduced chromatic aberration. The physical mechanism behind the FZP is that FZP produces a sequence of subsidiary foci around each major focus. These subsidiary foci of the FZP provide an extended depth of focus for each wavelength that partially overlaps with the other ones, creating an overall extended depth of focus that is less sensitive to chromatic aberration. However, the price paid to gain depth of field and reduce chromatic aberration is a reduced resolution, low energy efficiency, and strong background stray light^16^,^17^. In 2007, Andersen *et al*. proposed a broadband photon sieves telescope system consisting of an antihole photon sieves. However, in order to obtain a useful bandwidth, a complex corrective system that incorporates a second DOE and two extra mirrors with an even larger aperture than the photon sieves primary must be employed^18^. Recently, Zhao *et al*. demonstrated that broadband optical imaging of a photon sieves can be implemented in a relatively simple way by using wavefront coding, in which a separated bulk phase mask is placed in front of a conventional photon sieves (CPS) to reduce the sensitivity of photon sieves to incident wavelength^19^.

In this paper, we propose and demonstrate a novel diffractive photon sieves that works at broad bandwidth, in which the pinhole pattern in a photon sieves is wavefront coded and generates equal focusing power for a wide range of operating wavelength in a single thin-film element without any auxiliary elements. Experimental validation was performed using an UV-lithography fabricated wavefront coded photon sieves of a focal length of 500 mm and a diameter of 50 mm. Results show that the working bandwidth of the wavefront coded photon sieves reaches 28 nm compared with 0.32 nm of the conventional one. Further demonstration of the achromatic imaging with a bandwidth of 300 nm (full visible region) is also performed with a photon sieves integrated with a refractive lens. The proposed methodology of coding micro-structure pattern in a diffractive element breaks the limit of inherent wavelength-dependence in conventional diffractive elements and opens up new possibilities for manipulating wavefront phase and achieving new applications or new functionalities of diffractive optical elements.

## Wavefront coded photon sieves (WFCPS)

### Theory

**Figure 1:**
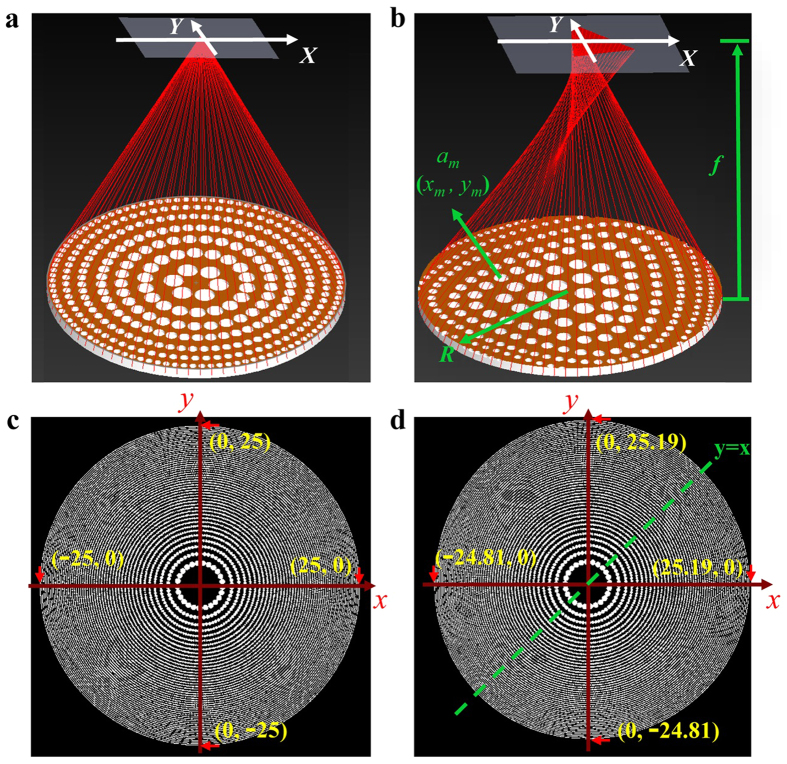
Schematic configurations of a CPS and a WFCPS with a coding parameter α=30π. (**a**) Schematic of a CPS and the ray path. (**b**) Schematic of a WFCPS with wavefront coding and the ray path. (**c**) Illustration of pinhole distribution of a CPS with an aperture of 50 mm, focal length of 500 mm at 632.8 nm. (**d**) Illustration of pinhole distribution of a WFCPS with the same geometrical dimensions as (**c**).

The geometry position of *m*th pinhole at *n*th ring in a wavefront coded photon sieves (WFCPS) is described by equation (1):


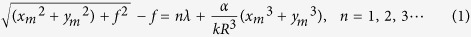


where *f* is the focal length, 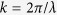 is the wave number, *λ* is the designed wavelength, *x*_*m*_ and *y*_*m*_ is the central location of the *m*th pinhole, *n* is an integer representing the sequential of the rings of the photon sieves, *R* is the radius of the photon sieves. It is noted that a cubic term is introduced in equation (1) that serves as the wavefront coding for the imaging photon sieves with *α* being a coding parameter (directly correlated to the operation bandwidth), as detailed in the supplementary information 1. Figure 1a shows a schematic of the circularly distributed pinhole arrangement of a conventional photon sieves (CPS), and Fig. 1b shows the coded pinhole pattern of the proposed WFCPS. The introduction of the cubic term modifies the symmetric pinhole arrangement in a CPS geometrically and thus alters the wavefront of the transmitted focusing light of the WFCPS such that different wavelengths over a wide range of bandwidth gain equal focusing power, and subsequently, achieving broadband achromatic operation with the WFCPS. From the point of view of point spread function (PSF) of an optical system, the introduction of the cubic term changes the dependence of the PSFs on the incident wavelength in which PSFs of the system at different wavelengths remain unchanged over a broadband incident wavelength, in contrast to the sensitive dependence of the PSFs on wavelength in a CPS. The flat behavior of the PSFs of a cubic phase modulated system on broadband wavelength renders the fundamental mechanism of broadband achromatic operation of a WFCPS.

To give a more detailed illustration of the theory, a CPS with an aperture of 50 mm, focal length of 500 mm is designed at wavelength of 632.8 nm. The total ring number of the photon sieves is 987 and the minimum pinhole size is 6.3282* μm* [Fig. 1c]. Positions of each pinhole of the CPS are calculated with equation (1) when the coding parameter α = 0. The pinhole sizes of the CPS are determined as described in supplementary information 2. A WFCPS with the same parameters (i.e., an aperture of 50 mm and a focal length of 500 mm) as that of the CPS is also designed with a coding parameter α = 30π (equation (1)). Unlike the structure of the CPS, in which coordinates of the outermost pinholes are symmetrically at (25 mm, 0 mm), (−25 mm, 0 mm), (0 mm, 25 mm) and (0 mm, −25 mm) on x- and y-axis, respectively, the structure of the WFCPS with a coding parameter α = 30π is a modified pattern in which the coordinates of the outermost pinholes are at (25.19 mm, 0 mm), (−24.81 mm, 0 mm) (0 mm, 25.19 mm) and (0 mm, −24.81 mm), respectively, showing symmetry along y = x [Fig. 1d].

### Simulations

**Figure 2:**
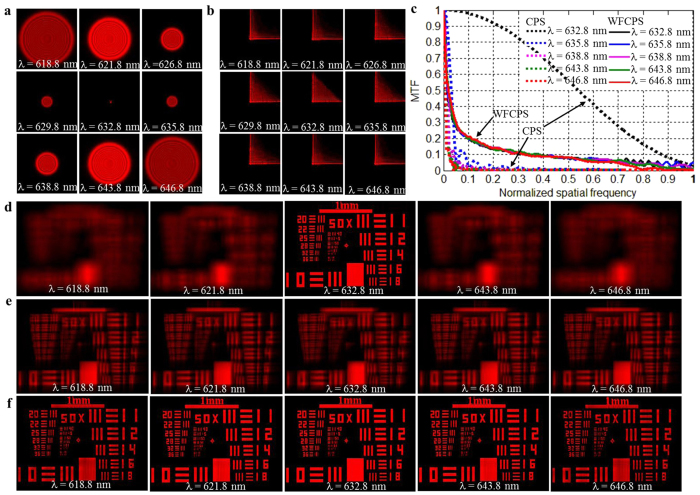
The simulated PSFs, MTFs and imaging behaviors of a CPS and a WFCPS at different wavelengths from λ=618.8 nm to 646.8 nm. (**a**) PSFs of a CPS. (**b**) PSF of a WFCPS with a coding parameter α = 30π. (**c**) MTFs of the corresponding PSFs in (**a,b**). (**d**) CPS imaging. (**e**) WFCPS intermediate imaging. (**f**) WFCPS restored imaging.

Figure 2a,b show the point spread functions (PSFs) of a CPS and a WFCPS with coding parameter α = 30π at different incident wavelengths ranging from *λ* = 618.8 nm to 646.8 nm. It is seen that the PSFs of the CPS is diffused with the increased wavelength deviation from the designed wavelength of 632.8 nm [Fig. 2a], which is understandable due to the high wavelength dependence of the diffractive elements. In contrast, the PSFs of the WFCPS remain almost unchanged within a wide range of the wavelengths from *λ* = 618.8 to 646.8 nm. This wavelength range represents the boundary of the consistency of the PSFs at different wavelengths, which can thus be considered as the operation bandwidth of the WFCPS.

The modulation transfer functions (MTF) that correspond to Fig. 2a,b are calculated by taking a Fourier transform of the PSFs and shown in Fig. 2c. It is consistent with those observed in Fig. 2a,b, MTFs drop significantly and zeros appear in the MTFs of the CPS when the incident wavelengths deviate from the designed one, resulting in the loss of spatial frequencies in the image. In contrast, MTFs are nearly identical in the case of WFCPS within the wavelength range from *λ* = 618.8 nm to 646.8 nm with slight deviation at high frequencies. Because of the consistency of the MTFs at different wavelengths in the WFCPS, and there is no zero points appeared in the MTFs, the wavefront coded blurred image at different wavelengths can be restored by using an appropriately designed digital filter. Hence the WFCPS can greatly reduce the sensitivity of the imaging device to the incident wavelength, resulting in extension of the working bandwidth of a photon sieves.

Figure 2d–f show the simulated imaging behaviors of the designed CPS and its comparison with those of the WFCPS at different wavelengths (*λ* = 618.8~646.8 nm). The images of the CPS become increasingly fuzzy when the incident wavelength deviates from the designed wavelength increasingly (Fig. 2d). The bandwidth of the CPS can be quantified by 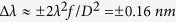 in our case^18^. Figure 2e,f show the blurred intermediate images and the corresponding restored images of the WFCPS, respectively. The image restoration is implemented with Wiener filtering technique^20^ in which the blurred intermediate images shown in Fig. 2e are deconvoluted with the filtering function of an averaged PSF from *λ* = 618.8 nm to 646.8 nm shown in Fig. 2b. It is seen that all the intermediate blurred images can be well restored with a fixed filtering function for all the wavelengths and all the restored images have almost the same resolution as that of the CPS in the designed wavelength, i.e., 632.8 nm in our case, with very small deviation at *λ* = 618.8 nm or 646.8 nm due to the small inconsistency of the MTF at large deviation from the designed wavelength. It should be mentioned that the blurred images of a CPS at wavelengths deviating from the designed one (i.e., 632.8 nm in our case) cannot be restored by using digital filtering method as used in WFCPS because of the fundamental limitation in filtering functions, as detailed in the supplementary information 3. In the case of a WFCPS with *D* = 50 mm, *f* = 500 mm, and α = 30π, the bandwidth can be as large as 28 nm, which implies that the extension of the bandwidth of the WFCPS is about two orders of magnitude higher than that of the conventional one.

### Experiments

The demonstration of the proposed WFCPS imaging is performed and compared with that of a CPS of the same numerical aperture and focal length. Supplementary information 4 shows the experimental arrangement and the fabricated photon sieves. A CPS with a focal length of 500 mm and a diameter of 50 mm at wavelength 632.8 nm and a WFCPS with coding parameter α = 30π and the same focal length and aperture as the CPS are fabricated using UV lithography.

**Figure 3:**
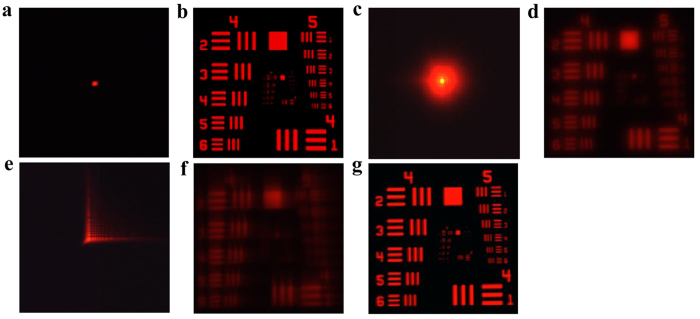
PSF and image measurements of a CPS and a WFCPS. (**a**) PSF of a CPS at single wavelength 632.8 nm. (**b**) Image of a CPS at single wavelength 632.8 nm. (**c**) PSF of a CPS with a broadband source of 28 nm bandwidth. (**d**) Image of a CPS with the 28 nm-bandwidth broadband source. (**e**) PSF of a WFCPS with broadband source of 28 nm bandwidth. (**f**) Intermediate blurred image produced by the WFCPS with the 28 nm-bandwidth broadband source. (**g**) Restored image of the WFCPS with 28 nm-bandwidth broadband source.

We first evaluate the performance of the CPS, respectively, under illumination of a single wavelength 632.8 nm and broadband source of bandwidth 28 nm. Figure 3a,b show the PSF measurements and the target images of the CPS with a diameter of 50 mm and focal length 500 mm under single wavelength 632.8 nm. The target used in the imaging experiment is USAF 1951. Detailed examinations show that the optical resolution of the CPS under single wavelength is about 50.8l p/mm (corresponding to 16* μm* focal spot), which is very close to the diffraction limited spot size of 15.44 *μm*. The effect of a broadband incident light on the image of the CPS is shown in Fig. 3c,d, which gives the PSF measurement result and the imaging result using a broadband light source centered at 632.8 nm with a FWHM bandwidth of 28 nm. No surprising, as a diffractive optical element, the CPS suffers from large chromatic aberration when a broadband incident light is used, in which a blurred PSF and a blurred image are generated (compared with the image in Fig. 3a,b). As a comparison, Fig. 3e–g show the PSF measurement and the imaging result of a WFCPS with a wavefront coding parameter α = 30π using a broadband light source. Figure 3e shows the experimentally measured PSF, which takes “L-shape”, same as those predicted in the simulation shown in Fig. 2b. Figure 3f shows the intermediate blurred image (i.e., before digital image filtering) produced by the WFCPS. The restoration of the wavefront coded image is shown in Fig. 3g. The restoration is performed using Wiener filtering technique in which the blurred intermediate image shown in Fig. 3f is deconvoluted with the filtering function of PSF shown in Fig. 3e. It is seen that the intermediate blurred image can be well restored and the restored image of broadband wavelength illumination show the almost same resolution as that of the CPS imaging at the designed single wavelength 632.8 nm, shown in Fig. 3b. It is estimated, from Fig. 3g, that the optical resolution of the WFCPS is at the level of 50.8 *lp/mm* using a broadband illumination of a FWHM bandwidth of 28 nm. Detailed comparison of the image quality between the CPS and WFCPS can be performed with the MTFs from the experimental imaging results shown in Fig. 3. The MTFs are calculated from the Fourier transform of the experimental images of Fig. 3 (see supplementary information 4 for details).

### Ultra-broadband (300 nm bandwidth) achromatic imaging with WFCPS

**Figure 4:**
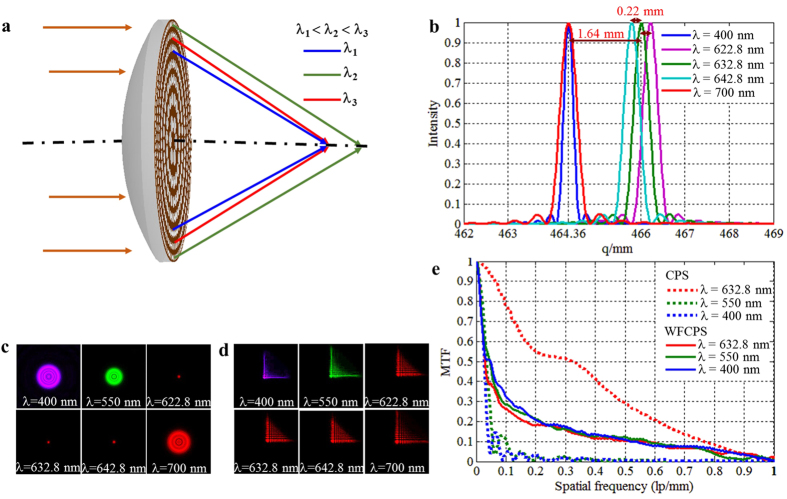
Hybrid achromatic element. (**a**) Schematic of a hybrid achromatic element and the ray path. (**b**) The diffractive field distributions of a designed conventional hybrid element along the optical axis with different illumination wavelengths. (**c**) PSFs of the conventional hybrid element at different wavelengths from 400 nm to 700 nm. (**d**) PSFs of an ultra-broadband wavefront coded hybrid element with a coding parameter α = 10π. (**e**) MTFs of the corresponding PSFs in (**c,d**).

There have been report of chromatic aberration correction of DOEs applying the method of hybrid refractive-diffractive^6^, the achromatic bandwidth is, however, limited within the translated wavelength of the depth of focus, typically 5–20 nm in the visible region. With a WFCPS integrated with a refractive element, the achromatic imaging bandwidth can be one order of magnitude higher than that conventional one. The chromatic aberrations of a DOE produce a longer focal length for shorter wavelengths (*λ*_*1*_) than that for longer wavelengths (*λ*_*3*_), while the chromatic aberrations of a refractive lens are opposite. One can thus combine the two optical effects to cancel chromatic aberration over a certain wavelength range. The simplest implementation involves fabrication of a DOE and a convex refractive surface on opposite sides of a single element (Fig. 4a). The focal lengths of two boundary wavelengths (*λ*_*1*_ and *λ*_*3*_) can be designed to be achromatic, however, the focal length difference (the secondary spectrum) between central (*λ*_*2*_) and boundary (*λ*_*1*_ and *λ*_*3*_) wavelengths is still retained, implying that large chromatic aberration of the system is still retained and consequently achromatic imaging would not be possible if the boundary wavelengths range is wide enough. With a designed WFCPS, the large chromatic aberration in the secondary spectrum can be eliminated resulting in ultra-broadband achromatic imaging.

#### Simulations

To illustrate the ultra-broadband achromatic imaging with WFCPS, a conventional diffractive and refractive hybrid element which combines a refractive lens with a CPS designed for a working bandwidth of 300 nm is given. Design parameters: focal length *f*  = 466 mm, aperture D = 50 mm, *λ*_*1*_ = 400 nm, *λ*_*2*_ = 632.8 nm, *λ*_*3*_ = 700 nm, and bandwidth (*λ*_*3*_ − *λ*_*1*_) = 300 nm. The refractive glass BK7 (refractive index 1.515 at 632.8 nm, Abbe number 28.9627) is adopted. According to supplementary information equation (S22), when the focal length of a CPS is 6864.8 mm and the focal length of a refractive lens is 499.954 mm at central wavelength 632.8 nm, the focal lengths are 464.36 mm, 466 mm and 464.36 mm at wavelengths *λ*_*1*_ = 400 nm, *λ*_*2*_ = 632.8 nm, and *λ*_*3*_ = 700 nm, respectively, which means that the focal lengths at boundary *λ*_*1*_ and *λ*_*3*_ are the same (achromatic). It is seen, however, a focal length difference of 1.64 mm still exists between focal lengths of the central wavelength *λ*_*2*_ and either of the boundary wavelength *λ*_*1*_ or *λ*_*3*_, as shown in Fig. 4b. This means that the conventional hybrid element cannot work achromatically in such a wide bandwidth of 300 nm. As a check, the depth of focus of the conventional hybrid element is 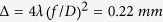 only, corresponding to wavelength 622.8 nm or 642.8 nm, a bandwidth of 20 nm only. The visual evidence of the limited achromatic bandwidth of the conventional hybrid element can also be seen in Fig. 4c, in which the PSF of the element remains within the diffraction limit between wavelength 622.8 nm and 642.8 nm, but diffuses when the working wavelengths are beyond the 20 nm range especially at both boundary wavelengths of 400 nm and 700 nm.

In contrast, When a WFCPS with a coding parameter α = 10π is integrated with a refractive lens with the same parameters as that in the conventional hybrid element mentioned above, a consistent PSF patterns can be obtained at all wavelengths ranging from 400 nm to 700 nm, Fig. 4d. The MTFs that correspond to Fig. 4c,d are calculated by taking a Fourier transform of the PSFs and are shown in Fig. 4e (only MTFs between 400 nm to central wavelength 632.8 nm are plotted due to the symmetrical nature). It is clear that MTFs of the wavefront coded element are nearly identical in the full visible range from *λ* = 400 nm to 700 nm while the MTFs of the conventional hybrid element drop significantly and zeros appear when the incident wavelengths deviate from the central wavelength 632.8 nm. The consistency of the MTFs in the wavefront coded element renders the possibility of achromatic imaging in an ultra-wide wavelength range.

**Figure 5:**
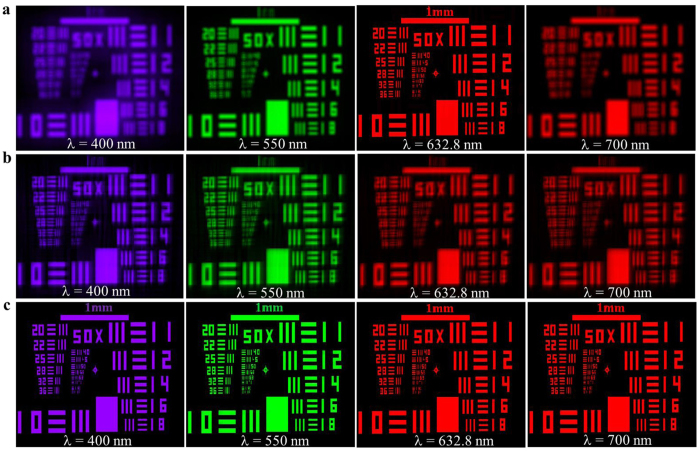
The simulated imaging behaviors of a conventional hybrid element and an ultra-broadband wavefront coded hybrid element at different wavelengths from 400 nm to 700 nm. (**a**) Conventional hybrid element imaging. (**b**) Wavefront coded hybrid element intermediate imaging. (**c**) Wavefront coded hybrid element restored imaging.

Figure 5a–c show the simulated imaging behaviors of the designed WFCPS element and its comparison with the conventional hybrid element at different wavelengths from *λ* = 400 nm to 700 nm. The images of the conventional hybrid element become increasingly fuzzy when the incident wavelength deviates from the central wavelength 632.8 nm increasingly (Fig. 5a). Figure 5b,c show the blurred intermediate images and the corresponding restored images of the wavefront coded element, respectively. It is seen that all the intermediate blurred images can be well restored with a fixed filtering function for all the wavelengths and all the restored images have almost the same resolution as that of the conventional hybrid element at the designed central wavelength, i.e., 632.8 nm in our case. With parameters *D* = 50 mm, *f* = 466 mm, and α = 10π, the achromatic imaging bandwidth of the wavefront coded element can be as large as 300 nm (could be even larger when coding parameter α is further optimized), covering the whole visible spectral range.

#### Experiments

**Figure 6:**
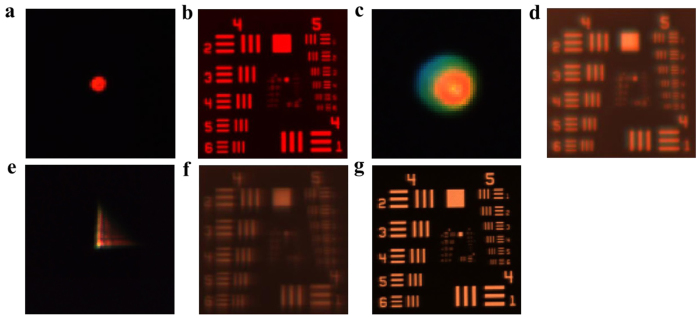
PSF and image measurements of a conventional hybrid element and a wavefront coded hybrid element. (**a**) PSF of a conventional hybrid element under illumination centered at 632.8 nm with a FWHM bandwidth of 28 nm. (**b**) Image of a conventional hybrid element under illumination of a 28 nm bandwidth. (**c**) PSF of a conventional hybrid element under illumination with a bandwidth of 300 nm. (**d**) Image of a conventional hybrid element under illumination of a 300 nm bandwidth. (**e**) PSF of a wavefront coded element under illumination of a 300 nm bandwidth. (**f**) Intermediate blurred image generated by the wavefront coded element under illumination of a 300 nm bandwidth. (**g**) Restored image of the wavefront coded element under illumination of a 300 nm bandwidth.

The demonstration of the proposed ultra-broadband achromatic imaging is performed and compared with that of a conventional hybrid element of the same numerical aperture and focal length. The wavefront coded element is formed with a UV fabricated WFCPS (α = 10π) on a plano-convex refractive lens while the conventional hybrid element is formed with a CPS on a plano-convex refractive lens with the same parameters (*D* = 50 mm) as that of wavefront coded one. The performance of the conventional hybrid element, under illumination of a bandwidth of 28 nm and an ultra-wide bandwidth of 300 nm (from 400 nm to 700 nm), respectively, are firstly evaluated. Figure 6a,b show the PSF measurement and imaging of the conventional hybrid element with a diameter of 50 mm and focal length 466 mm under light source centered at 632.8 nm with a FWHM bandwidth of 28 nm. It is seen that the image within the bandwidth of 28 nm is acceptable (although the bandwidth of illumination is slight larger than the designed 20 nm working bandwidth of the element). The performance of the conventional hybrid element under illumination of 300 nm bandwidth is shown in Fig. 6c,d, in which the PSF measurement and the imaging are given. No surprising, the conventional hybrid element suffers from large chromatic aberration, in which a blurred PSF and a blurred image are clearly observed.

As a comparison, Fig. 6e–g show the PSF measurement and the imaging result of a wavefront coded element with a coding parameter α = 10π using a broadband light source from 400 nm to 700 nm. Figure 6e shows the experimentally measured PSF, and Fig. 6f shows the intermediate blurred image (i.e., before digital filtering) generated by the wavefront coded element. Fig. 6g gives the achromatic imaging with a bandwidth of 300 nm after restoration of the intermediate blurred image. The optical resolution of the wavefront coded element under 300 nm bandwidth is better than that of conventional hybrid element under 28 nm bandwidth, and is the same as that of conventional hybrid element at the single central wavelength 632.8 nm. Detailed comparison of the image quality between the conventional hybrid element and wavefront coded element is performed with the MTFs and given in supplementary information 6.

## Conclusion

We propose and demonstrate a novel achromatic imaging with diffractive photon sieves that works in broad bandwidth covering the whole visible spectral range. The pinhole pattern in a photon sieves is wavefront coded and generates equal focusing power for a wide range of operating wavelength in a single thin-film element without any auxiliary elements. Experimental validation was performed using an UV-lithography fabricated wavefront coded photon sieves of a focal length of 500 mm and a diameter of 50 mm. Results show that the working bandwidth of the wavefront coded photon sieves reaches 28 nm with a selected coding parameter α = 30π compared with 0.32 nm of the conventional one. Further demonstration of the achromatic imaging with an ultra-wide bandwidth of 300 nm (in whole visible region from 400 nm to 700 nm) is also performed with a wavefront coded photon sieves integrated with a refractive lens. It should be noted that the working bandwidth could be further extended when the wavefront coding parameter α is further optimized by taking into account the trade-off between the bandwidth and the acceptable SNR. The proposed methodology of coding micro-structure pattern in a diffractive element breaks the limit of inherent wavelength-dependence in conventional diffractive elements and opens up new possibilities for manipulating wavefront phase and achieving new applications or new functionalities of diffractive optical elements.

## Methods

### Optical simulations

Figure 2a,b show the point spread functions (PSFs) of a CPS and a WFCPS with a coding parameter α = 30π, which are calculated based on supplementary information equation (S17) using MATLAB computing program at different incident wavelengths ranging from *λ* = 618.8 nm to 646.8 nm. The modulation transfer functions (MTF) that correspond to Fig. 2a,b are calculated by taking a Fourier transform of the PSFs and are shown in Fig. 2c. Figure 2d–f show the simulated imaging behaviors of the designed CPS and its comparison with those of the WFCPS at different wavelengths (*λ* = 618.8~646.8 nm). The simulated images of the photon sieves are obtained by a convolution calculation between a target object (i.e., a patterned test plate) and the PSFs shown in Fig. 2a,b. All the simulations are performed using MATLAB computing program.

### Photon sieves fabrication

Experimental photon sieves were fabricated using UV-lithography. High transmittance glass substrate in visible region is double-side polished and cleaned. A 300 nm thick chromium layer is deposited on the substrate. A layer of positive photoresist is spin-coated on the chromium surface and the thickness of the photoresist is 500 nm. The photoresist layer is exposed to the ultraviolet laser beam (351 nm), and the photon sieves structure is obtained after development of the photoresist layer using 8‰ NaOH. The photon sieves structure in the photoresist layer forms a mask and then the structure is transferred from the photoresist layer to the chromium layer by a liquid chemical agent, containing glacial acetic acid of 3.35 ml, deionized water of 1 L, and ceric ammonium nitrate of 200 g, removing the chromium layer on the substrate in the areas that are not protected by photoresist. Finally, the remaining photoresist layer is removed by 1% NaOH.

### Ultra-broadband achromatic imaging element fabrication

Firstly, a plano-convex refractive lens of center thickness 4.3 mm is fabricated and the refractive glass BK7 is adopted. A photon sieves can be fabricated using UV-lithography on the flat surface of a plano-convex refractive lens to form a hybrid achromatic element. A 300 nm thick chromium layer is deposited on the flat surface and a layer of positive photoresist is spin-coated on the chromium surface and the thickness of the photoresist is 500 nm. The other steps are the same as the photon sieves fabrication.

## Additional Information

**How to cite this article**: Zhao, X. *et al*. Ultra-broadband achromatic imaging with diffractive photon sieves. *Sci. Rep.* **6**, 28319; doi: 10.1038/srep28319 (2016).

## Supplementary Material

Supplementary Information
