# Supplementary material for: Ultra-broadband achromatic imaging with diffractive photon sieves
Source: Sci Rep. 2016 Jun 22;6:28319. doi: 10.1038/srep28319 (PMC4916432; doi:10.1038/srep28319)
Supplement: Supplementary Information [file srep28319-s1.doc]

**Ultra-broadband achromatic imaging with diffractive photon sieves**

**Xiaonan Zhao^1,2^, Jingpei Hu^1,2^, Yu Lin^1,2^, Feng Xu^1,2,*^, Xiaojun Zhu^3^, Donglin Pu^1,2^, Linsen Chen^1,2^, & Chinhua Wang^1,2,*^**

^1^College of Physics, Optoelectronics and Energy & Collaborative Innovation Center of Suzhou Nano Science and Technology, Soochow University, Suzhou 215006, China.

^2^Key Lab of Advanced Optical Manufacturing Technologies of Jiangsu Province & Key Lab of Modern Optical Technologies of Education Ministry of China, Soochow University, Suzhou 215006, China.

^3^School of Electronics and Information, Nantong University, Nantong 226019, Jiangsu, China

Corresponding author: [xf750617@suda.edu.cn](mailto:xf750617@suda.edu.cn); [chinhua.wang@suda.edu.cn](mailto:chinhua.wang@suda.edu.cn)

**S1: Design principle of a wavefront coded photon sieves**

**Cubic phase wavefront coding:** It is noted that a cubic term is introduced in Eq. (1) that serves as the wavefront coding for the imaging photon sieves with *α* being a coding parameter. Through the use of the ambiguity function^1^ and the stationary-phase method^2^, the cubic wavefront coding for a broadband achromatic photon sieves imaging are readily found. The ambiguity function is an analytical tool that permits us to observe and design optical transfer function (OTF) of a system. The stationary-phase method provides the analytical flexibility needed to consider only wavefront coding in this design process.

The photon sieves is a diffractive optical element (DOE) and, as such, suffers from dispersion. With a focal length *f* of a photon sieves, the images at other wavelengths will be defocused relative to the designed wavelength *λ*. The focal length will change with wavelength according to^3^

$\frac{\Delta f}{f}=\frac{\Delta\lambda}{\lambda}$ (S1)

where Δ*λ* is wavelength deviation and Δ*f* is defocus. The defocus coefficient W_20_ is dependent on the photon sieves size as well as the focus state:

$\frac{2\pi}{\lambda}W_{20}=\frac{\pi D^{2}}{4\lambda}\left(\frac{1}{f}-\frac{1}{d_{o}}-\frac{1}{d_{i}}\right)$ (S2)

where *D* is the diameter of the photon sieves aperture, *d_o_* is the object distance and *d_i_* is the image distance. In the case of plane wave illumination, Eq. (S2) can be given by:

$\frac{2\pi}{\lambda}W_{20}=\frac{\pi D^{2}}{4\lambda}\left(\frac{1}{f}-\frac{1}{f+\Delta f}\right)$ (S3)

The defocus Δ*f* of the photon sieves can be approximated as:

$\Delta f=8W_{20}\left(\frac{f}{D}\right)^{2}$ (S4)

Combining Eq. (S1) with Eq. (S4), one can obtain the relationship between the defocus coefficient W_20_ and the wavelength deviation Δ*λ*:

$W_{20}=\frac{\Delta\lambda\cdot D^{2}}{8\lambda f}$ (S5)

Consider a one-dimensional unit-power phase function, in normalized coordinates, such as:

$P\left(x\right)=\left\{\begin{matrix} \frac{1}{\sqrt{2}}\exp\left[j\theta\left(x\right)\right] , \left|x\right|\leq1 \\ 0 , \mathrm{otherwise} \end{matrix}\right.$ (S6)

where *j* is the imaginary unit and we assume that *θ* (*x*) is a monomial:

$\theta\left(x\right)=\alpha x^{\gamma}, \gamma\neq\left\{0, 1\right\}, \alpha\neq0$ (S7)

α is called coding parameter. The ambiguity function can be used as a polar display of the OTF^4^, given by:

$H\left(u,W_{20}\right) =A\left(u,v\right)=\frac{1}{2}\int_{-\left(1-\left|u\right|/2\right)}^{\left(1-\left|u\right|/2\right)} \exp\left\{j\left[g\left(x\right)+2\pi vx\right]\right\}dx, \left|u\right|\leq2$ (S8)

where *u* is the spatial frequency, $\nu\text{=}2uW_{20}/\lambda$, and $g\left(x\right)=\alpha\left[\left(x+u/2\right)^{\gamma}-\left(x-u/2\right)^{\gamma}\right]$.

If the phase term *g*(*x*) varies fast enough, the above integral can be approximated through the stationary point of $\left[g\left(x\right)+2\pi vx\right]$. The stationary-phase approximation for *A* (*u, v*) is given by:

$A\left(u,v\right)\approx\frac{1}{2}\left[\left|\frac{\partial x_{i}}{\partial v}\right|\right]^{1/2}\exp\left\{j\left[2\pi vx_{i}+g\left(x_{i}\right)\right]\right\}$ (S9)

From approximation Eq. (S9) the magnitude of the ambiguity function will be independent of its second parameter *v* when stationary point *x_i_* is linear in *v*. In order to find stationary point *x_i_*, we can begin by taking the derivative of $\left[2\pi vx_{i}+g\left(x_{i}\right)\right]$ and setting the result equal to zero. We obtain:

$\partial\left[2\pi vx_{i}+g\left(x_{i}\right)\right]/\partial x_{i}=2\pi v+\gamma\alpha\left(x_{i}+u/2\right)^{\gamma-1}-\gamma\alpha\left(x_{i}-u/2\right)^{\gamma-1}=0$ (S10)

We can show that the solution for *x_i_* above, as a function of *γ*, will be linear in *v* if and only if:

$\gamma=3$ (S11)

The needed wavefront coding modified term will then have a cubic phase modulation. This cubic phase modulation has a stationary point of

$x_{i}=\frac{-\pi v}{3\alpha u}, u\neq0$ (S12)

Then the OTF of the WFCPS can be given by:

$H\left(u,W_{20}\right) \text{=}A\left(u,2uW_{20}/\lambda\right)\approx\left(\frac{\pi}{12\left|\alpha u\right|}\right)^{1/2}\exp\left(j\frac{\alpha u^{3}}{4}\right)\exp\left[-j\left(\frac{2\pi}{\lambda}W_{20}\right)^{2}\frac{u}{3\alpha}\right], u\neq0$ (S13)

Substituting Eq. (S5) into Eq. (S13), one can obtain the relationship of the OTF and the wavelength deviation Δ*λ*:

$H\left(u,\Delta\lambda\right) \approx\left(\frac{\pi}{12\left|\alpha u\right|}\right)^{1/2}\exp\left(j\frac{\alpha u^{3}}{4}\right)\exp\left[-j\left(\frac{\pi D^{2}}{f\lambda^{2}}\right)^{2}\frac{\left(\Delta\lambda\right)^{2}u}{48\alpha}\right], u\neq0$ (S14)

From (S14), it is clear that the magnitude of the OTF (MTF) is independent of the wavelength deviation Δ*λ.* The phase term affects only the shifting of the location of the resulting PSF with large wavelength deviation, which can be suppressed with large values of α. The final approximation for the OTF is then

$H\left(u,\Delta\lambda\right) \approx\left(\frac{\pi}{12\left|\alpha u\right|}\right)^{1/2}\exp\left(j\frac{\alpha u^{3}}{4}\right), u\neq0$ (S15)

**Discussions on the coding parameter:** The effect of the coding parameter α on the performance of the broadband achromatic imaging of a WFCPS includes the consistency of MTFs and the signal-to-noise ratio of the system.

The consistency of MTFs of the WFCPS system (focal length 500mm, diameter 50mm, designed wavelength 632.8nm) at different wavelengths can be evaluated by the mean squared error (MSE) between the MTF_Δ_*_λ_* of the WFCPS at wavelength deviation Δ*λ* and the MTF_0_ of the WFCPS at designed wavelength:

$MSE=\sqrt{\frac{\sum_{i=1}^{N} \left(MTF_{\Delta\lambda}-MTF_{0}\right)^{2}}{N}}$ (S16)

where *N* is the sampling points at different spatial frequencies. According to equation (S14) and equation (S16), Figure S1 shows the MSE of the WFCPS at wavelength deviation Δ*λ*=14nm for coding parameters ranging from α=0 to α=90π. It is easy to see from Fig. S1 that the larger the coding parameter α, the lower MSE, i.e., the less sensitive to wavelength the MTF, which means a wider operation bandwidth of the WFCPS.


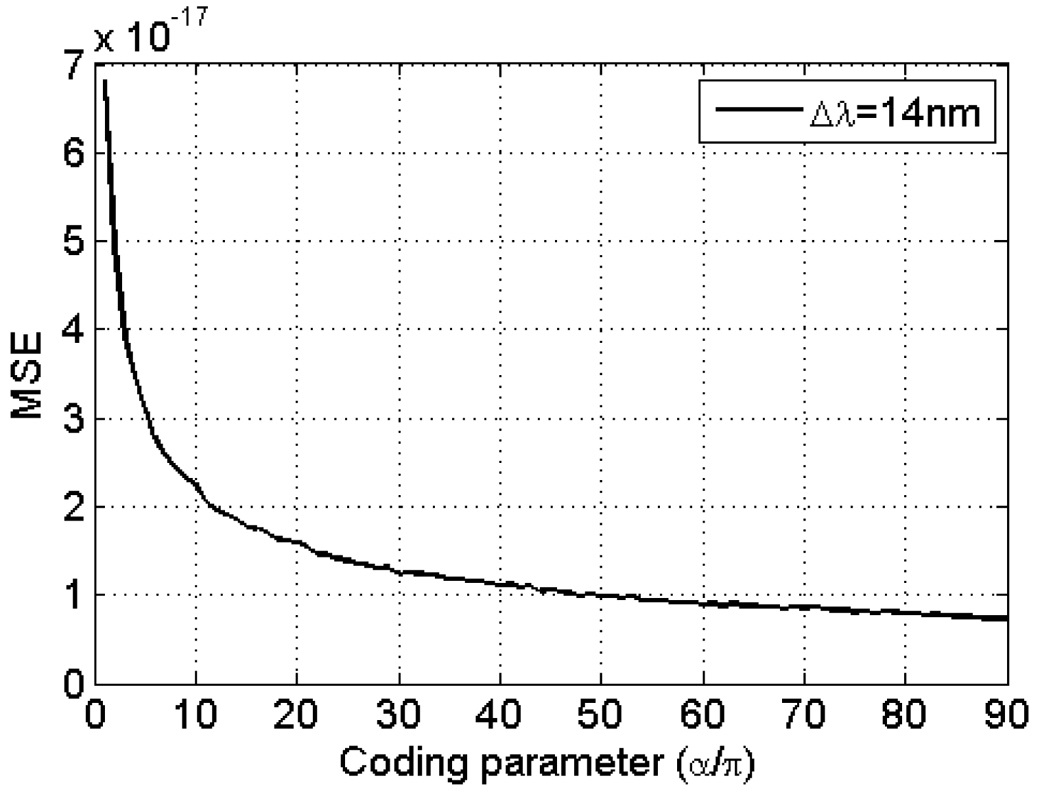


**Figure S1  MSE of a WFCPS as a function of coding parameter with a wavelength deviation Δ*λ*=14nm**

On the other hand, when the coding parameter α increases, the MTFs of the system decreases (Figure S2), which means an increased noise level during the process of deconvolution, and subsequently a decreased signal-to-noise ratio (SNR) of the system.

From Fig. S1 and S2, it is seen that a trade-off between maximal operation bandwidth and acceptable image SNR must be made with an appropriate coding parameter α. In practical, the coding parameter is usually between 5π - 50π depending on specific purpose.

**
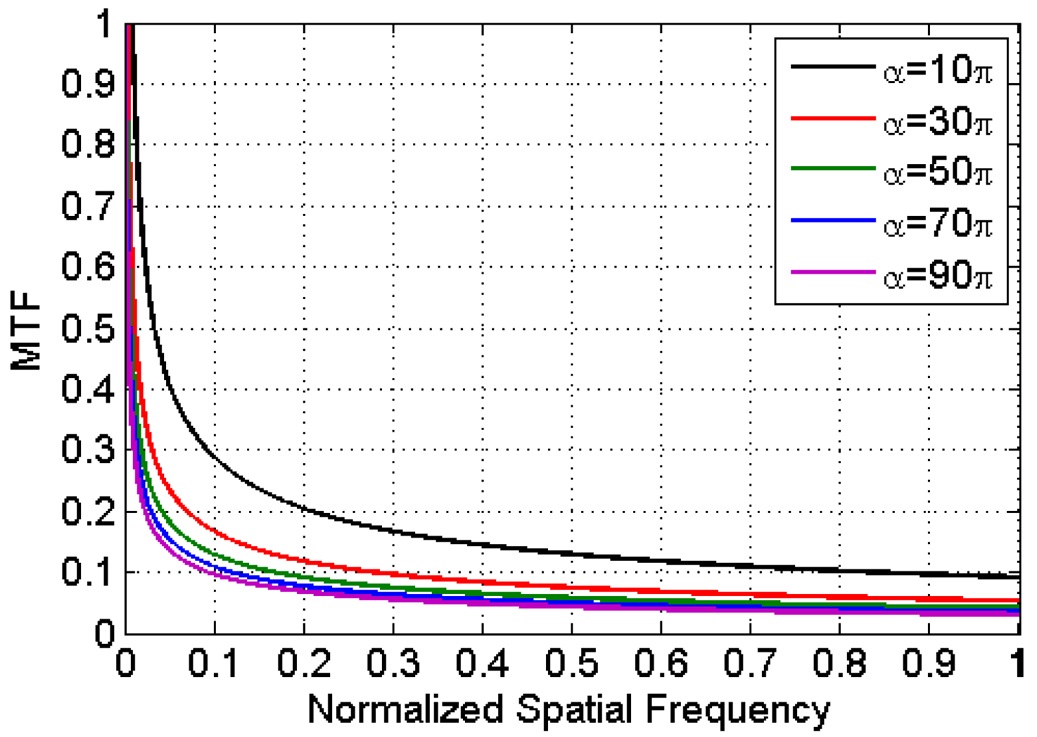
**

**Figure S2  MTFs of a WFCPS (Eq. (S14)) at different coding parameters with a wavelength deviation Δλ=14nm**

**S2: The total diffracted field distribution of a photon sieves**

The total diffracted field distribution of a photon sieves at the focal plane is a summation of those individual diffracted fields from different pinholes^5–7^. According to the individual far-field model^7^, one can obtain the diffractive field at the image plane:

$U\left(X,Y\right)\text{=}\sum_{m=1}^{M} U_{m}\left(X,Y\right)=\sum_{m=1}^{M} \frac{kA_{m}a_{m}^{2}q}{H^{2}}\exp\left[jk\left(L_{m}+H\right)\right]Jinc\left(\frac{ka_{m}}{H}\rho\right)$ (S17)

where$U_{m}\left(X,Y\right)$is the individual diffracted field from the *m*th pinhole, *M* is the number of pinholes, $k=2\pi/\lambda$ is the wave number, *λ* is the wavelength, *A_m_* is real amplitude and *L_m_* is the eikonal of the illuminative beam at the photon sieves, *j* is the imaginary unit, *a_m_* is the radius of the pinhole, *q* is the distance between the photon sieves and the image plane, $H=\left[q^{2}+\left(X-x_{m}\right)^{2}+\left(Y-y_{m}\right)^{2}\right]^{\frac{1}{2}}$, $\rho=\left[\left(X-x_{m}-g_{m}H\right)^{2}+\left(Y-y_{m}-h_{m}H\right)^{2}\right]^{\frac{1}{2}}$, *x_m_* and *y_m_* is central location of the *m*th pinhole, $g_{m}=\left(\frac{\partial L}{\partial x}\right)\left|_{x_{m},y_{m}}\right.$, $h_{m}=\left(\frac{\partial L}{\partial y}\right)\left|_{x_{m},y_{m}}\right.$, and $Jinc\left(\cdot\right)=\frac{J_{1}\left(\cdot\right)}{\left(\cdot\right)}$ is the *Jinc* function and $J_{n}\left(\cdot\right)$ is the *n*th-order Bessel function of the first kind.

The determination of the pinhole size *a_m_* of the *m*th pinhole at *n*th ring in Eq. (S17) can be made as follows: A photon sieves is essentially evolved from a Fresnel zone plate in which the rings at a radial distance *r*_n_ in the Fresnel zone plate have been replaced by isolated circular holes at the same radial distance *r*_n_ in the photon sieves. The width *w_n_* of *n*th ring in a Fresnel zone plate can be represented by the following equation (S18) such that the area of each ring is a constant^7^:

$w_{n}=\frac{\pi f}{kr_{n}}$ (S18)

where $k=2\pi/\lambda$is the wave number, *λ* is the wavelength, *f* is the focal length, and *r*_n_ is the radial distance of the *n*th ring of the zone plate.

The diameter of the pinholes at the corresponding *r*_n_ (i.e., *n*th ring) can be calculated based on the diffracted field at the center of the imaging plane of the *m*th pinhole at *n*th ring, *U_m,n_*(0, 0), which can be represented by the following oscillating function^5,7^,

$U_{m,n}\left(0,0\right)\propto\frac{d_{n}}{w_{n}}J_{1}\left(\frac{\pi d_{n}}{2w_{n}}\right)$ (S19)

where *J*_1_ is a first-order Bessel function. When *U_m,n_*(0, 0)>0, light passing through the pinholes is making a positive contribution to the focused light, while *U_m,n_*(0, 0)<0 the transmitted light is acting to reduce the focused intensity. From equation (S19), it is seen that positive contribution to the focused light can be obtained with pinhole diameters *d_n_* between*=*0 to 2.4*w_n_* or 4.4*w_n_* to 6.4*w_n_*, and so on, determined by the oscillating nature of the first-order Bessel function. In our design, we choose *d_n_*=*w_n_* at *n*th ring for simplicity, and *w_n_* is given by Eq. (S18).

**S3: Restoration of the blurred images in a CPS**

The blurred image in the case of a CPS when the wavelength deviates from the designed one cannot be restored by using the same method as that in a WFCPS or other filtering method. The fundamental physical mechanism is that when incident wavelengths deviate from the designed one, the MTFs of the system drop rapidly and zeros appear in the MTFs of the CPS, resulting in the loss of spatial frequencies in the image, as shown in Fig. 2c. If we try to restore the blurred image of the CPS with a point spread function (PSF) that corresponds to the information-lost MTFs of the CPS shown in Fig. 2c, no restoration of images can be obtained. Figure S3 shows the “restored” images of the CPS at different wavelengths including the designing wavelength 632.8nm. The “restored” images at different wavelengths are obtained by Wiener filtering technique (same method as that used in WFCPS) in which the images shown in Fig. 2d at different wavelengths are deconvoluted with the corresponding filtering function of the PSFs from λ=618.8nm to 646.8nm in Fig. 2a.

It is seen that all the “restored” images at deviated wavelengths from the designed one (632.8nm) are even worse than that without restoration. At the designed wavelength 632.8nm, the quality of the “restored” image is similar to that without restoration shown in Fig. 2d. This is expected, because “bad” filtering functions (i.e., PSFs shown in Fig. 2a) are utilized in the process of the restoration, in which high spatial frequencies information are lost, as witnessed by the corresponding MTFs shown in Fig. 2c.


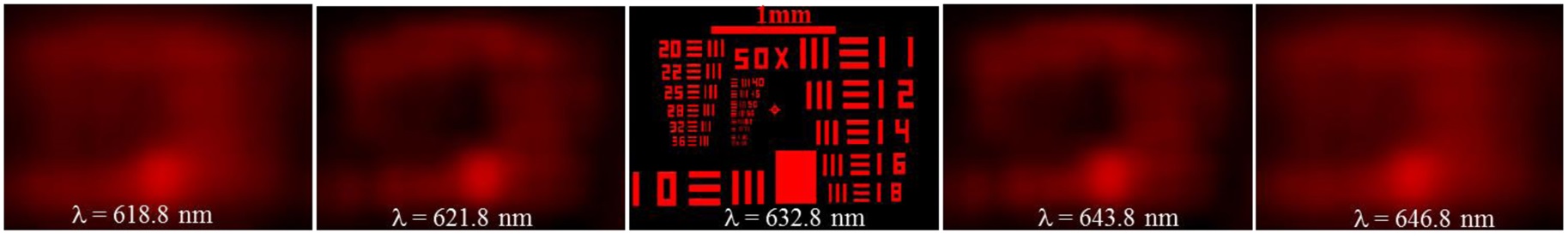


**Figure S3  Restored images of the CPS at different wavelengths**

**S4: Experimental imaging of a CPS and a WFCPS**

Figure S4 shows the experimental arrangement. A CPS with a focal length of 500mm and a diameter of 50mm at wavelength 632.8nm and a WFCPS with coding parameter α=30π and the same focal length and aperture as the CPS are fabricated using UV lithography. The photos of the fabricated CPS and WFCPS are shown in the inset of Fig. S4, respectively. A He-Ne laser and a tungsten lamp are switchable, with and without the mirror, for the single wavelength and broadband imaging, respectively. The bandwidth of broadband source can be controlled with a bandpass filter. Inset of Fig. S4 shows the spectrum of a bandpass filter with central wavelength at 632.8nm and a full width at half magnitude (FWHM) of 28nm (Semrock, FF02-632/22-25). The bandwidth of 300nm is obtained with a long-pass filter of cut-off wavelength 400nm (Thorlabs, FELH0400) and a short-pass filter of cut-off wavelength 700nm (Thorlabs, FESH0700). A collimator with a focal length 550mm, and a diameter 55mm is used to generate a collimated beam. A CCD with pixel size 4.54*μm* (AVT Prosilica GX2750C) is used.


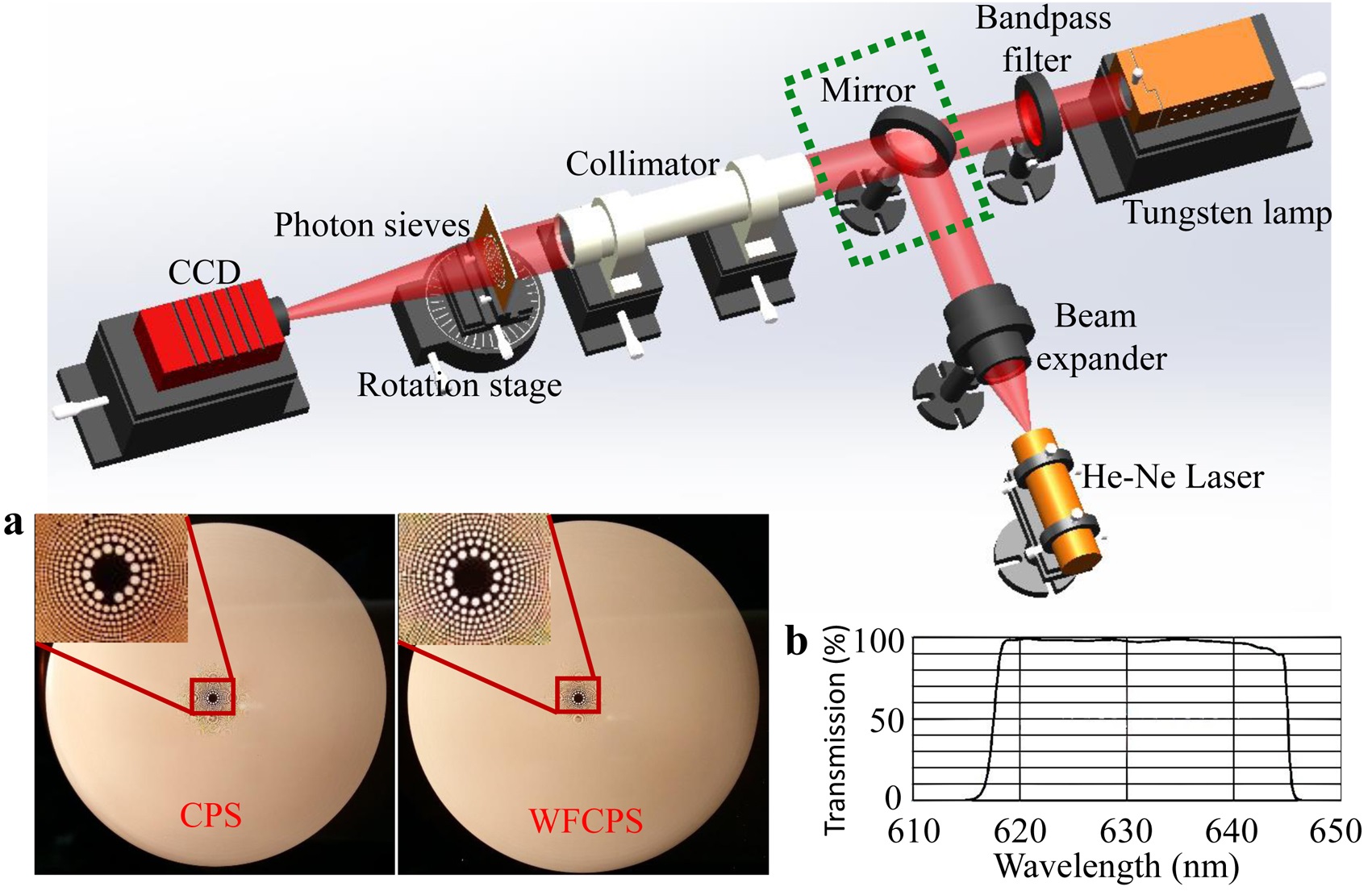


**Figure S4  Experimental setup with switchable single wavelength and broadband imaging. Insets: a,** a fabricated CPS and WFCPS. **b,** Transmission curve of a bandpass filter.

Figure. S5 shows respectively the MTFs of the CPS imaging at single wavelength 632.8nm (dotted line) and a broadband wavelength of FWHM=28nm (circle line), as well as the MTF of the image with the WFCPS (solid line) under broadband illumination. As expected, MTF drops rapidly and zeros appear in the MTF (circle line) when the CPS is illuminated with a broadband source, which is consistent with the observation in Fig. 3c and d. In contrast, the MTF (solid line) of the wavefront coded imaging under broadband illumination is similar to that of the CPS at single wavelength 632.8nm (dotted line), which provides a quantitative evidence for the observed experimental images. It should be noted that the working bandwidth could be further extended when the wavefront coding parameter α is further optimized. It is known that the larger the coding parameter α, the less sensitive to wavelength the PSF/MTF, and consequently, the lower the MTF, which results in the noise enhancement after deconvolution. The effect of the phase parameter α on the working bandwidth and the image signal-to-noise ratio (SNR) can be optimized with the help of the calculation power of modern image processing and also the criteria of image SNR, to achieve a trade-off between maximal bandwidth and acceptable image SNR.


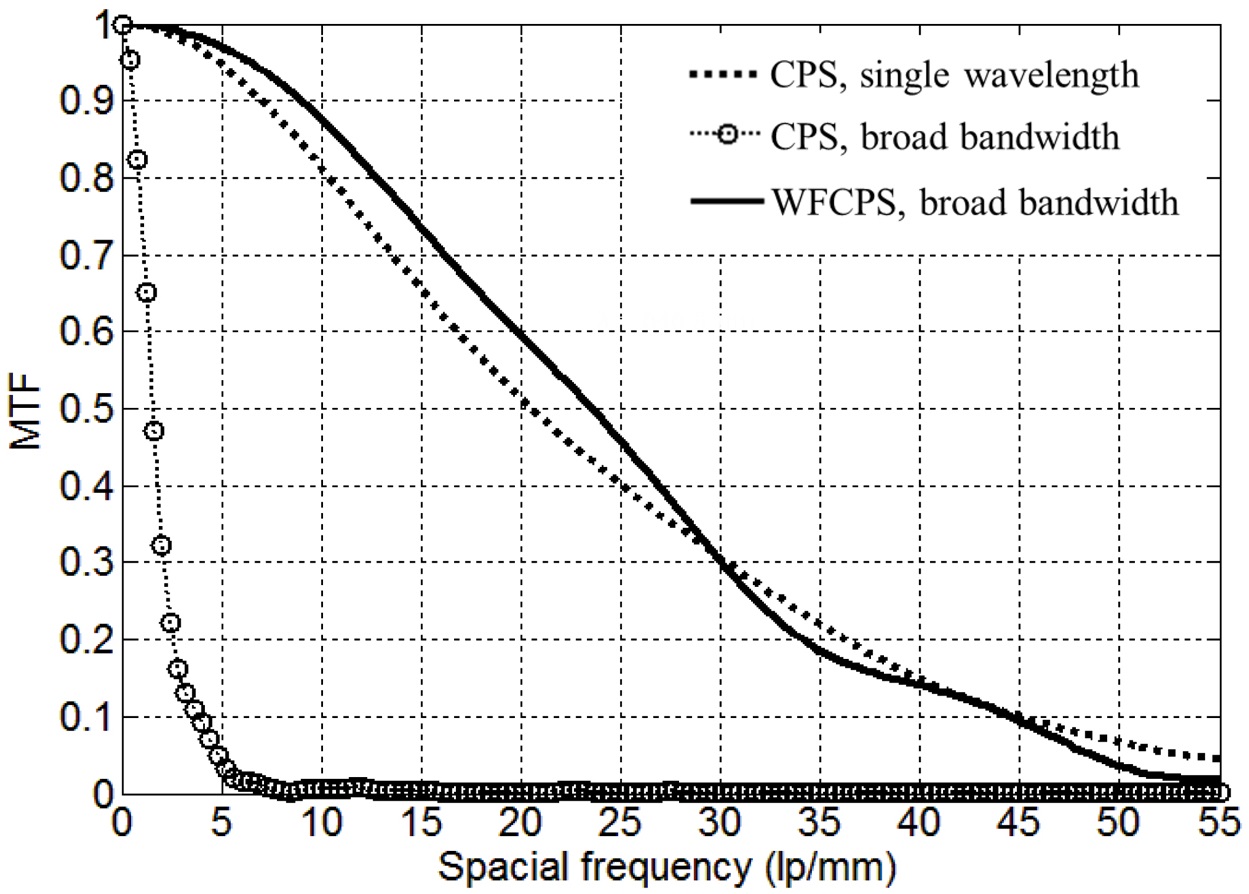


**Figure S5  A quantitative evaluation of the experimental images.** MTF curve of the CPS imaging at single wavelength (dotted line) and broadband wavelength (circle line), as well as the WFCPS imaging (solid line) at broadband wavelength.

**S5: Design method of a hybrid achromatic element**

The effective Abbe number of a DOE is described by:

$V_{DOE}\text{=}\frac{\lambda_{2}}{\lambda_{1}-\lambda_{3}}$ (S20)

The Abbe number of a refractive lens is described by:

$V_{\text{re}f}\text{=}\frac{n_{2}-1}{n_{1}-\text{n}_{3}}$ (S21)

We can obtain the focal length distribution at central wavelength λ_2_:

$\left\{\begin{matrix} f_{DOE\_2}=f\times\frac{V_{DOE}-V_{ref}}{V_{DOE}} \\ f_{ref\_2}=f\times\frac{V_{ref}-V_{DOE}}{V_{ref}} \end{matrix}\right.$ (S22)

where *f DOE_2* is the focal length of a DOE, *f ref_2* is the focal length of a refractive lens and *f* is the focal length of the hybrid achromatic element at central wavelength λ_2_.

**S6: A quantitative evaluation of experimental imaging quality of a conventional hybrid element and a wavefront coded element**

Figure. S6 shows respectively the MTFs of the conventional hybrid element imaging with a bandwidth of 28nm (dotted line) and 300nm (circle line) illumination, as well as the MTF of the image with the wavefront coded element (solid line) under illumination of 300nm bandwidth. As expected, MTF drops rapidly and zeros appear in the MTF (circle line) when the conventional hybrid element is illuminated with a broadband source, which is consistent with the observation in Fig. 6c and d. The MTF of the image with 28nm bandwidth illumination (dotted line) is close to the diffraction limit (slight lower than the diffraction limit because the bandwidth of illumination is slight larger than the 20nm working bandwidth of the element). In contrast, the MTF (solid line) of the wavefront coded imaging under 300nm bandwidth illumination is very close to the diffraction limit, which provides a quantitative evidence for the observed experimental images.


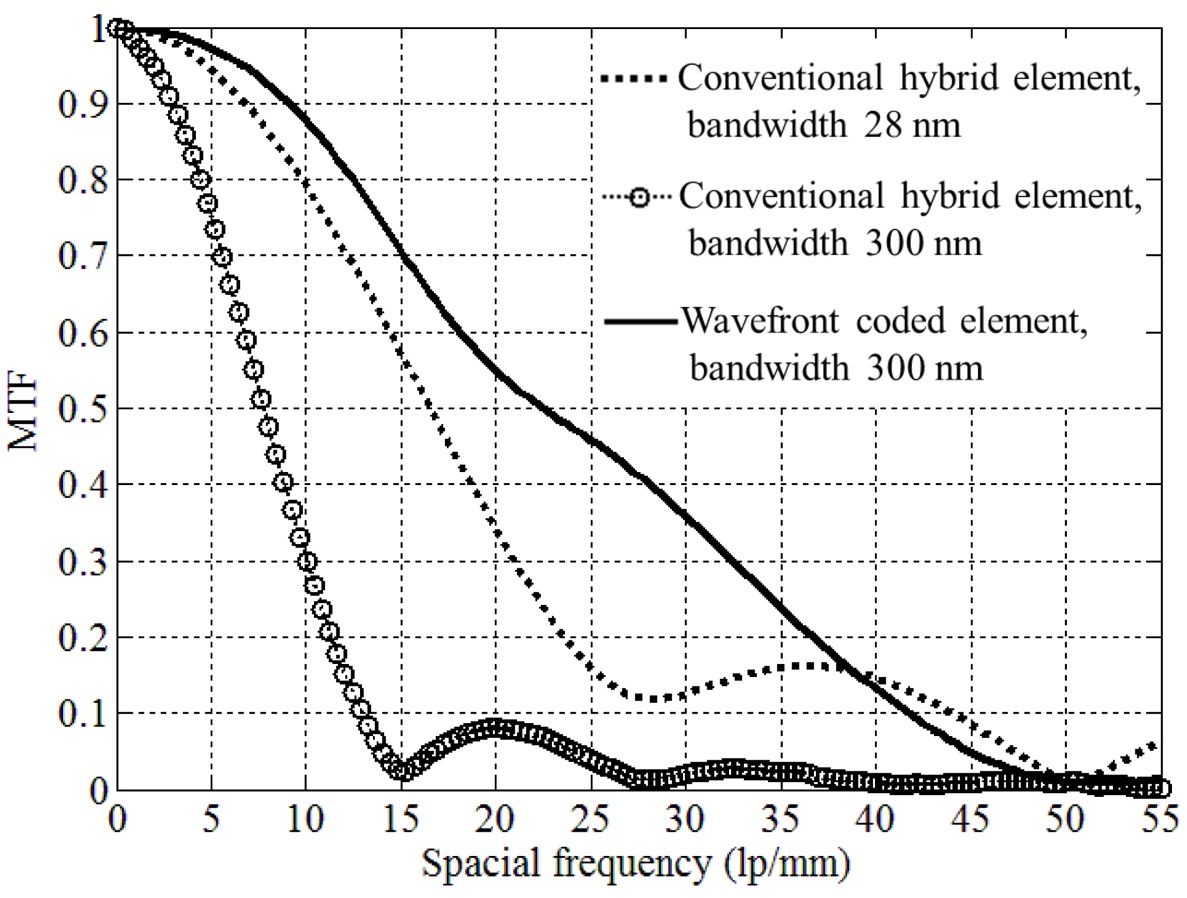


**Figure S6  A quantitative evaluation of the experimental images.** MTF curve of a conventional hybrid element under a bandwidth of 28nm (dotted line) and 300nm (circle line), as well as the MTF of the wavefront coded element imaging (solid line) under a bandwidth of 300nm.

**Supplementary References**

1. Brenner, k.-H., Lohmann, A. W. & Ojeda-Castaneda, J. The ambiguity function as a polar display of the OTF. *Opt. Commun*. **44**, 323–326 (1983).
2. Born, M. & Wolf, E. Principles of Optics, 6th edn (Cambridge University Press, 2002).
3. Andersen, G. & Tullson, D. Broadband antihole photon sieve telescope. *Appl. Opt.* **46**, 3706–3708 (2007).
4. Dowski, E. R. & Cathey, W. T. Extended depth of field through wave-front coding. *Appl. Opt.* **34**, 1859–1866 (1995).
5. Kipp, L. *et al*. Sharper images by focusing soft X-rays with photon sieves. *Nature* **414**, 184–188 (2001).
6. Cao, Q. & Jahns, J. Focusing analysis of the pinhole photon sieve: individual far-field model. J. Opt. Soc. Am. A **19**, 2387–2393 (2002).
7. Cao, Q. & Jahns, J. Nonparaxial model for the focusing of high-numerical-aperture photon sieves. J. Opt. Soc. Am. A **20**, 1005–1012 (2003).
